# Testing the Effect of Medical Positive Reinforcement Training on Salivary Cortisol Levels in Bonobos and Orangutans

**DOI:** 10.1371/journal.pone.0108664

**Published:** 2014-09-24

**Authors:** Verena Behringer, Jeroen M. G. Stevens, Gottfried Hohmann, Erich Möstl, Dieter Selzer, Tobias Deschner

**Affiliations:** 1 Department of Primatology, Max Planck Institute for Evolutionary Anthropology, Leipzig, Germany; 2 Centre for Research and Conservation, Royal Zoological Society of Antwerp, Antwerp, Belgium; 3 Department of Biomedical Sciences/Biochemistry, University of Veterinary Medicine, Vienna, Austria; 4 Working Group for Wildlife Biology, Justus-Liebig-University Gießen, Gießen, Germany; University of Rennes 1, France

## Abstract

The management of captive animals has been improved by the establishment of positive reinforcement training as a tool to facilitate interactions between caretakers and animals. In great apes, positive reinforcement training has also been used to train individuals to participate in simple medical procedures to monitor physical health. One aim of positive reinforcement training is to establish a relaxed atmosphere for situations that, without training, might be very stressful. This is especially true for simple medical procedures that can require animals to engage in behaviours that are unusual or use unfamiliar medical devices that can be upsetting. Therefore, one cannot exclude the possibility that the training itself is a source of stress. In this study, we explored the effects of medical positive reinforcement training on salivary cortisol in two groups of captive ape species, orangutans and bonobos, which were familiar to this procedure. Furthermore, we successfully biologically validated the salivary cortisol assay, which had already been validated for bonobos, for orangutans. For the biological validation, we found that cortisol levels in orangutan saliva collected during baseline conditions were lower than in samples collected during three periods that were potentially stressful for the animals. However, we did not find significant changes in salivary cortisol during medical positive reinforcement training for either bonobos or orangutans. Therefore, for bonobos and orangutans with previous exposure to medical PRT, the procedure is not stressful. Thus, medical PRT provides a helpful tool for the captive management of the two species.

## Introduction

Over the past few decades, the management of captive animals has been improved in a variety of ways. One particularly effective element was the establishment of specific enrichment programs that contributed to the improvement of the well-being of captive individuals [1]. Another one concerns the establishment of positive reinforcement training (PRT) as a tool to facilitate interactions between caretakers and animals during examinations [1]. PRT has long been part of care and management in marine mammals such as dolphins and sea lions [2], [3] and has since then been applied more and more to the management of terrestrial animal species in zoos and laboratories [3].

By definition, PRT relies on the cooperation of the animal. It is a form of operant conditioning in which the animal gets a reward (e.g., food) from the trainer after showing the desired behavior (e.g., [4]–[6]. That is why PRT is also named reward training [2]. Often a handheld clicker is used as a conditioned or secondary reinforcer connecting the acoustic signal with the positive, or primary, reinforcer (e.g., food) [7]. PRT with a clicker is commonly used in different animal species and has become a common training method in nonhuman primates [5], [8], [9]. This kind of training gives the animal the choice to cooperate or not [4], and the training of medical procedures is a helpful tool in monitoring physical health [10]–[12].

PRT has become a valuable tool for scientific, veterinary and husbandry procedures and it is recommended by a number of professional guidelines (e.g., [13] or the “Guide of the Care and Use of Laboratory Animals” mentioned by Perlman et al. [14]). It has been carried out in a variety of species including birds [2], big cats [10], and bears [4]. PRT has been particularly successful when applied in the management of captive non-human primates (prosimians [15], New World monkeys [8], [14], [16], Old World monkeys [5], [17], and great apes [18]–[20] including chimpanzees [21]–[23], gorillas [24]–[26], bonobos [27]–[29], and orangutans [30]–[32]; for a review see Prescott and Buchanan-Smith [33]).

Common husbandry procedures in great apes include, for example: presentation of parts of the body [1], [18], [27], hand injections or venepuncture [27], [34]–[36], collection of blood [35]–[37], collection of urine [1], [24], [36], [38] as well as nail trimming, taking of body temperatures or body weight, and monitoring of heart and respiratory function (reviewed in [12]). In great apes, training has also been used to manage behavior, for example to improve maternal skills in gorillas [25], [39], bonobos [28] and orangutans [40].

PRT techniques, used as a part of nonhuman primate behavioral management programs, can be beneficial for both trainers and animals. For example, PRT can prevent stress during veterinary procedures and/or increase cooperation in research procedures [1], [14], [34]. It is widely believed that the quality of these interactions during PRT positively affects the well-being of the animal ([41], reviewed in [42]) for example by giving the animals more control over their environment (e.g., [4], [5], [34]). Therefore, PRT is considered to be beneficial and to improve the lives of animals through increased welfare and reduced stress [6]. However, for PRT to be successful, a trustful, close and safe relationship between keepers and the animals has to be established [27].

One aim of conducting PRT is to establish a relaxed atmosphere that facilitates animal care that, without the training, might be stressful [34], [35]. At the same time, one cannot exclude the possibility that the training itself is a source of stress because, for example, training interferes with other ongoing activities; it exposes subjects to unusually close proximity with human care givers and – in group-living species – may induce competition for attention of the trainer or for access to the reward [43]. On the other hand, group training (in which animals are not separated for training) may have the advantage that individuals can learn from each other, thereby increasing the effectiveness of medical PRT. Furthermore, separating animals for PRT can be stressful, especially for individuals of gregarious species [3], [44]. In this study, stress was defined as the response of the hypothalamo-pituitary-adrenal (HPA) axis to a stressor, resulting in an increase in cortisol in saliva (for a review see [45]).

To investigate if medical PRT could reduce stress hormone levels in comparison to otherwise necessary invasive examinations, previous studies have used blood or serum to measure levels of corticosteroids and found that PRT reduced stress during husbandry procedures like caging [46], venepuncture [47], hand injections [34] or blood sampling [9]. While these studies showed that PRT can help to reduce stress in husbandry procedures, the effects of PRT itself on stress in the animals in the training process has so far received little attention. Moreover, using blood to evaluate stress can be problematic, because the collection of these samples itself could result in cortisol increase [48]–[50]. Furthermore, blood cannot always be obtained easily even in a captive setting. While long-term stress responses can be measured non-invasively in primates from urine and fecal samples ([51], [52] but see [53] for potential pitfalls), saliva is more accurate to assess short-term stress response, as salivary cortisol levels reflect changes in serum cortisol levels with a delay of only five minutes [54]. Furthermore, saliva can be obtained relatively easily from apes in zoo settings using PRT, and has been used in bonobos [55]–[57], orangutans [58], gorillas [26] and chimpanzees [59]. PRT in lab-housed baboons did not lead to an increase in salivary cortisol levels [60]. However, the task the animals were trained for in this study was simple and included neither extensive trainer –animal interactions nor direct physical contact. Furthermore, the PRT was conducted with separated animals. Therefore it remains to be tested whether PRT with tasks including medical procedures that necessitate touching the animals, such as the measurement of body temperature from the ear or monitoring of heart and respiratory functions, would be perceived as stressful by the subjects.

In this study we assessed the effects of medical PRT on salivary cortisol in group training sessions in two species of captive apes: orangutan and bonobo. All animals were familiar and well experienced with the medical PRT. Therefore, the study design investigated the extent to which well-trained animals experience medical PRT to be stressful. In bonobos, salivary cortisol response has already been biologically validated and was shown to increase during periods of stress [55], [56]. For orangutans, a study had already measured salivary cortisol [58], but this study showed results of a single individual. Therefore, we first did a biological validation for salivary cortisol by comparing cortisol levels in saliva collected during baseline conditions, with samples collected during three periods that were potentially stressful for the animals [61]; in the next step we used the biologically validated salivary cortisol measurements to assess the influence of medical PRT on stress hormone levels, both in orangutans and bonobos, by comparing cortisol levels in samples collected during medical PRT with baseline levels. Furthermore, in bonobos, we compared training with half of the group with training with the whole group to investigate if the number of bonobos participating simultaneously in the PRT influenced the stress response.

## Methods

### Ethics Statement

Saliva collection is a non-invasive method. It was carried out in accordance with NIH published standards and the protocol of sample collection was approved by authorities of Frankfurt Zoo, Germany (Dr. Thomas Wilms). Study was approved by committee of Frankfurt Zoo (Dr. Rüdiger Dmoch and Dr. C. R. Schmidt 30^th^ May 2006).

Saliva samples were collected from a group of seven Sumatran orangutans and a group of ten bonobos in Frankfurt Zoo (table 1). Both species lived in social groups at all times; food was offered at least three times a day and consisted mainly of a mixture of fruits and vegetables. Animals had *ad libitum* access to fresh water. All subjects had access to indoor and outdoor enclosures. All apes had nearly continuous access to the indoor enclosures; access was restricted only during periods of cleaning. The enclosures contained natural substrates or concrete, climbing structures, and many manipulable objects (e.g., fire hose, ropes, wood-wool).

**Table 1:** Species, name of the individual, sex, age, and number (N) of saliva samples collected pre, during and post medical positive reinforcement training for salivary cortisol measurements.

Species	Name	Sex	Age	Training samples (N)		
				pre	During	post
Bonobo	Heri	M	7	4	4	4
Bonobo	Kelele	M	4	3	3	3
Bonobo	Ludwig	M	24	3	3	3
Bonobo	Haiba	F	7	3	4	3
Bonobo	Kamiti	F	21	2	3	3
Bonobo	Kutu	F	10	3	3	3
Bonobo	Magrit	F	57	3	3	3
Bonobo	Natalie	F	42	3	2	3
Bonobo	Ukela	F	23	3	3	3
Bonobo	Zomi	F	10	3	3	3
Orangutan	Charly	M	51	6	5	6
Orangutan	Galdikas	M	8	7	5	7
Orangutan	Lucu	M	3	7	5	7
Orangutan	Djambi	F	49	7	5	7
Orangutan	Jahe	F	5	7	5	7
Orangutan	Rosa	F	19	6	5	6
Orangutan	Sirih	F	16	7	5	7

Individuals of both groups had already participated previously in medical PRT sessions. The bonobo group was familiar with medical PRT since 1999 and PRT of the orangutans started in 2000. Training for these apes for saliva collection procedure is described in detail in Behringer et al. [55]–[57]. Apes were trained by PRT to chew on cotton rolls, and to return the chewed cotton for a reward.

Every medical PRT session included body examination, oral inspection, nail trim, measurement of body temperature from the ear, and monitoring of heart and respiratory function for every individual per session. No new tasks were introduced during the session in which saliva samples were collected. Training was carried out by the same keeper using preferred fruits as a primary reinforcement and a hand-held clicker as secondary reinforcement. Each orangutan and bonobo participated in every session, and individuals were trained in a random order. Generally, medical PRT could be conducted at any time of the day. However, samples for this study were taken at the same time of day to minimize the influence of diurnal patterns of salivary cortisol on the results. Since medical PRT in combination with sample collection was always interspersed between routine medical PRT, the anticipation of the training session by the apes was avoided. To have comparable situations for this study design, each training session lasted exactly 20 min. Although participation in the training sessions was voluntary and apes were not forced to approach the medical PRT place or to cooperate with the trainer, all individuals joined all training sessions. Training sessions started with the collection of a saliva sample. For the orangutans, in the first two training sessions, samples were only collected at the beginning and at the end. In the next five sessions, saliva samples were taken at the start (pre), after 10 min. (during) and again after 20 min. (post). The first sample was used as a baseline sample for each individual and served as a control for the samples taken after 10 and 20 min. [54]. After 10 min. of training, every individual had been involved at least once in the training. Although it was not possible to count the time each individual spent in each training session, the trainer ensured that every individual was trained in each procedure. For bonobos, in the first and third sessions, all ten individuals were trained together in one group. In the second and fourth sessions, the group was split up to explore whether group size has an effect on salivary cortisol concentration. The separation of the bonobos into two groups occurred during the morning hours to avoid the possible influence of the group split on salivary cortisol levels. Individuals could freely choose between two enclosures and thereby group composition was decided upon by the animals themselves. In all four sessions, samples were collected before, during and after medical PRT. In all sessions, 92 saliva samples were collected for bonobos.

### Orangutan saliva sampling protocol

For the orangutan group, two samples sets were collected. Sample set 1: To investigate the validity of salivary cortisol as a marker for stress response, we compared samples collected during potentially stressful situations (N = 50) with samples collected under baseline conditions (N = 68). Sample set 2: Multiple saliva samples were collected from the same individual during seven different training sessions (for details see table 1 and 2).

**Table 2:** Number (N) of orangutans and saliva samples for each condition.

Condition	Animals (N)	Samples (N)
Baseline	7	68
Stress event 1 (catch event)	7	17
Stress event 2 (day of transfer)	7	21
Stress event 3 (new enclosure)	7	12

#### 1. Baseline values and stress conditions

For baseline conditions, 68 salivary cortisol samples from the orangutan group were randomly chosen from a large sample set collected between December 2006 and December 2008. On these days, neither stressful events occurred nor was medical PRT performed. Samples were collected either during normal saliva collection at 13:00 (38 samples) or at 16:00 (30 samples). To investigate cortisol secretion under stress conditions in orangutans we chose three potential stress events. During these events, stress indicating behavior patterns such as yawning and self-scratching increased in frequency [61]. A) The capture of a three-year-old female orangutan on 22^nd^ December 2006. The young female was separated from the group at 12:45 and captured for medical reasons at 13:10. After 20 min. she was released back into the group. Samples were collected from all group members including the caught female, first before the stress event at 11:00, then during the event at 13:00 and finally directly after the event at 14:00. B) Orangutans were transferred into a new great ape house on 13^th^ May 2008. Orangutans were trained before to enter a transport cage and were therefore not anesthetized. Transfer into the sleeping boxes of the new ape house started at 8:30 and at 13:00 the new inside orangutan enclosure was opened for the whole group. The 21 saliva samples were collected whenever possible between 8:30 and 17:30. C) The third sample set was collected on the day following the transfer (first day in the new enclosure) at 13:00 and 16:00.

#### 2. Training conditions

To test the short term effect of medical PRT on salivary cortisol in the orangutan group, we collected saliva samples of all orangutans before and during seven routine training sessions performed with the whole group. All training sessions were carried out between 19^th^ February and 24^th^ April 2008 and started at 14:00±10 min. This time window for medical PRT was chosen for two reasons: First, to avoid interference with feeding times and therefore with food, which may affect measurements of enzymes and hormones in saliva [62] but see also [63]. Second, midday was chosen for sample collection to minimize confounding effects of diurnal variation of salivary cortisol [58], [64].

### Bonobo saliva sampling protocol

In a previous study we found that in response to socially stressful events such as transfer to a new facility or introduction of new group members, salivary cortisol of captive bonobos increased compared to baseline cortisol levels [56]. In the present study we collected saliva samples before, during and after medical PRT to compare the effect of the training with baseline before training. Sample material was collected during four routinely performed training sessions conducted between 26^th^ March and 10^th^ April, 2008 around 14:00 (same time and season as described for the orangutans).

### Sample preparation and cortisol assay

Samples were frozen immediately after collection at −20°C and shipped to the Department of Biomedical Sciences, University of Veterinary Medicine, Vienna, Austria. The frozen cotton rolls containing saliva were thawed and centrifuged (1500 g, 10 min.). While the use of cotton rolls produced artificial high testosterone values their use is unproblematic for cortisol measurements [59], [65]. Three microliters of the resulting saliva plus 47 µl of assay buffer were used for the cortisol assay. Cortisol was measured with an enzyme immunoassay (EIA) previously described by Palme and Möstl [66]. Validation of the EIA and measurement procedure are described in Behringer et al. [56], [61]. Intra- and inter-assay coefficients of variation of high and low value quality controls were 8.6% and 14.5% (N = 37) and 9.6% and 13.8% (N = 93), respectively. Samples were re-measured if bindings were outside of a 30–70% range (linear range of the assay) or if divergence of concentration duplicates was greater than 10%.

### Statistical analysis

We conducted three General Linear Mixed Models (GLMM) [67] using the function ‘lmer’ of the R-package lme4 [68], [69]. The first GLMM was run to investigate the effects of potentially stressful situations on salivary cortisol levels in the orangutan group. The second GLMM was used to determine the influence of medical PRT on salivary cortisol levels in orangutans, and the third GLMM to test the effects of medical PRT on salivary cortisol levels in the bonobo group. In all three models approximate normality and homogeneity of residuals was assessed by visual inspection of residuals plotted against fitted values and a qq-plot. All model assumptions were met. To achieve this, the response variable ‘cortisol’ was log-transformed in all three cases. To check for absence of collinearity, we examined Variance Inflation Factors (VIF [70]) using the function vif of the R-package car [71] applied to a standard linear model excluding random effects. These indicated that collinearity was not an obvious issue (maximum VIF; model 1: 1.13; model 2 (orangutan): 1.05; model 3 (bonobo): 1.19). Also, in all three models, the time of sample collection was z-transformed to a mean of zero and a standard deviation of one to achieve comparable estimates [72].

In the first GLMM we tested the influence of potentially stressful situations on salivary cortisol in orangutans. Four different stress events were included as a fixed effect, categorical predictor (with the levels: baseline conditions, catch, transfer, day after transfer). We also included sex, age, and time of sampling as predictors with fixed effects in the model and animal was included as a random intercept term. Sex and individual were included in the models because stress hormone levels may vary with sex and age [73]. To establish the significance of the fixed effects as a whole, we compared the full model with a null model excluding all fixed effects but retaining the random effects using a likelihood ratio test [74]; R function ‘ANOVA’). In order to achieve reliable P-values for the individual effects we used Markov Chain Monte Carlo sampling (MCMC) [67].

In the second and third model, the influence of medical PRT on the response salivary cortisol levels was investigated for the two data sets of orangutans and bonobos, respectively. Animal and session ID were included as random intercept terms in both models. The time of sample collection and age (z-transformed) were included as fixed effects, categorical predictor (with the levels pre, during and post medical PRT) as well as session number and sex. Furthermore, we also included random slopes of time of sample collection within animal into the model [75]. The random slope of time of sample collection within animal was not significant in orangutans (Chisq = 0, df = 3, P = 1) and bonobos (Chisq = 0, df = 2, P = 1), indicating that the effect of time of sample collection was not significant different across animals. Also we did a full null model comparison for both models, as described above for model 1. Tests of significance were calculated using MCMC as described above. To establish significance for the overall effect of treatment (pre, during, or post medical PRT) we used the functions ‘pvals.fnc’ and ‘aovlmer.fnc’, as provided by the R package languageR [76]. Significance for all tests was set at the 0.05 level.

## Results

To investigate the possible influence of the fixed effect in the three different models on salivary cortisol, we compared the full model with a null model in both species. These comparisons revealed significant effects for each species and models (model 1: P<0.001; model 2 (orangutan): P = 0.01983; model 3 (bonobo): P = 0.005).

### Orangutans

#### Comparison of orangutan salivary cortisol levels in stressed and baseline conditions

**Figure 1:**
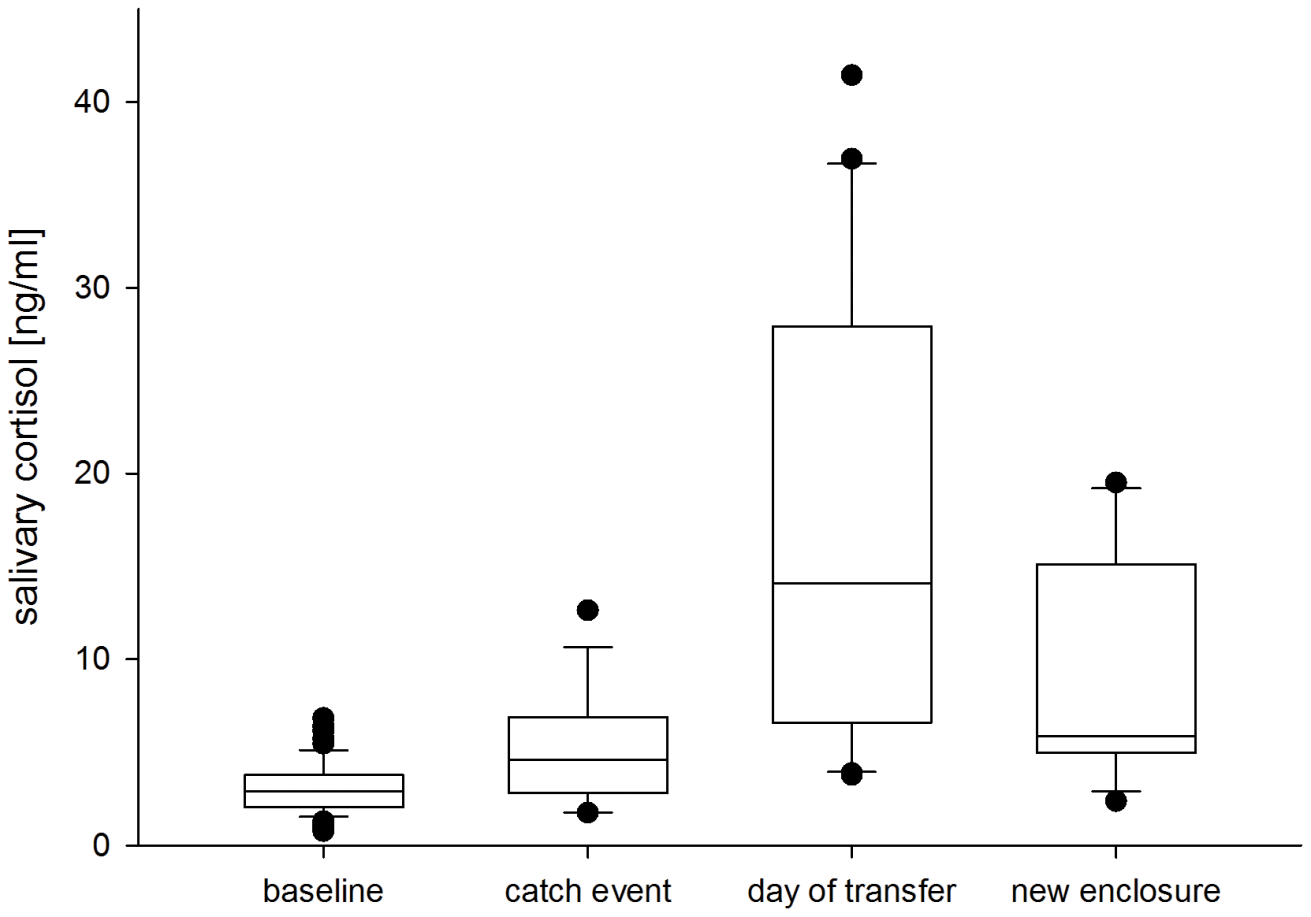
Average salivary cortisol concentration in orangutans for baseline levels and for the three potentially stressful events (catch event, day of transfer, and new enclosure). The boxes illustrate the 25th and 75th percentiles, bars indicate medians, and circles indicate outliers.

We compared cortisol levels in orangutan saliva under baseline conditions (median 2.92 ng/ml, SD. 1.34 ng/ml) and potentially stressful situations (median 6.59 ng/ml, SD. 10.0 ng/ml). When subjects were exposed to potentially stressful events (inducing all three tested stress events), salivary cortisol levels were elevated compared to the baseline condition (overall test of the effect of the factor: P_MCMC_<0.001, Figure 1). To investigate whether all three events affect salivary cortisol levels, we conducted a post-hoc comparison. For each of the three potentially stressful events, salivary cortisol levels were significantly higher compared to the baseline condition (Table 3), with the effect being most pronounced on the day of the transfer (Table 3). Sex, age, and time of sample collection did not have a significant effect on salivary cortisol levels (Table 3).

**Table 3:** Results of the General Linear Mixed Models of the subset obtained from orangutans when exposed to stress and at baseline conditions with salivary cortisol as response variable (sampling time (z-transformed), age and sex were included as fixed effects and animal ID was included as random intercept term). Bold values indicate P<0.05.

	Estimate	Std. Error	P_MCMC_
Intercept	0.9255	0.086	
Sex	0.1224	0.109	0.348
Time of sampling	0.1026	0.059	0.184
Age	0.0228	0.054	**0.712**
Stress event 1	0.5577	0.161	**<0.001** †
Stress event 2	0.9816	0.181	**<0.001** †
Stress event 3	1.6832	0.159	**<0.001** †

† comparing the events to baseline conditions.

#### Salivary cortisol levels during medical PRT in an orangutan and bonobo group

**Figure 2:**
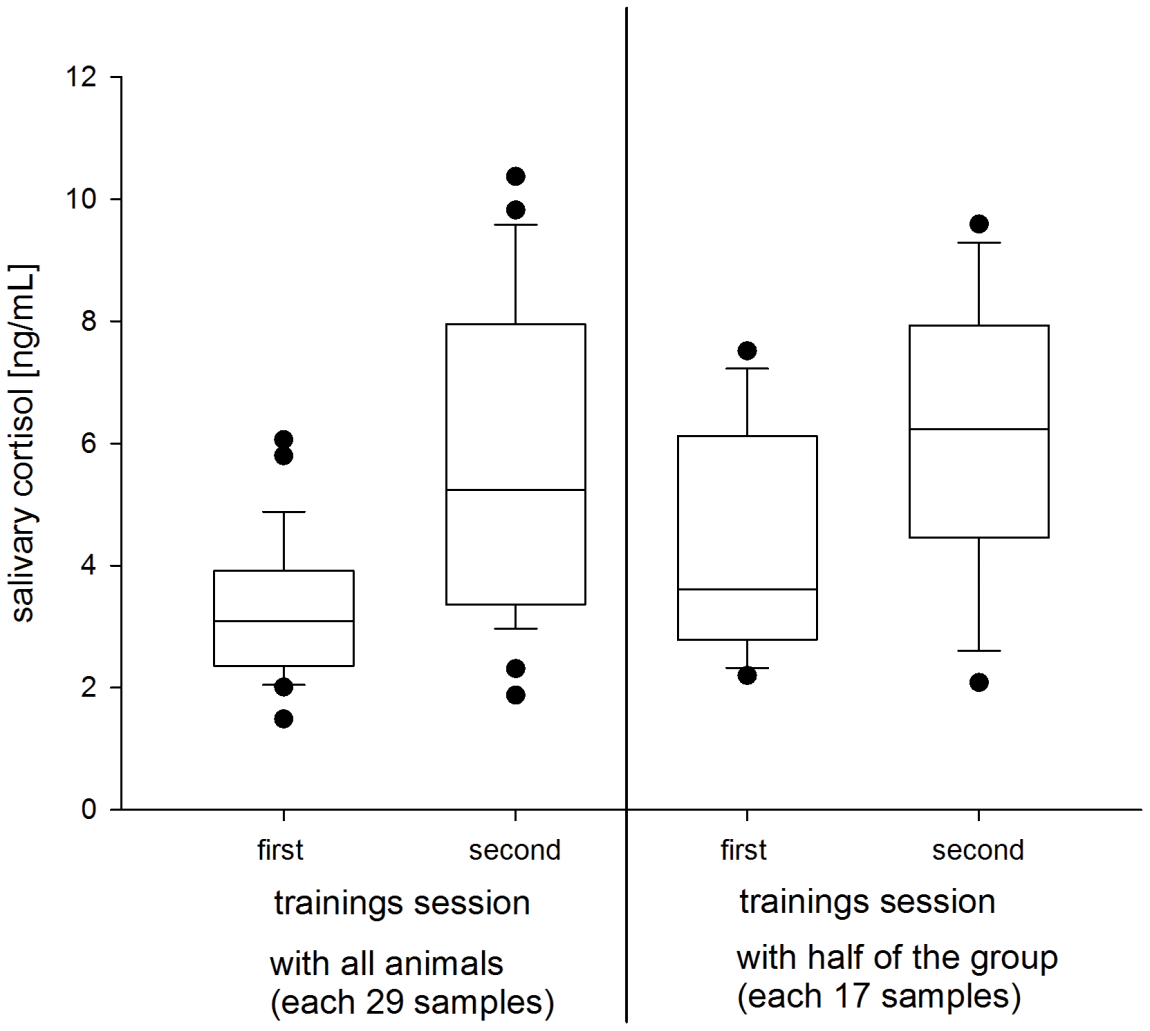
Average salivary cortisol concentration of males and females of a bonobo group during four medical PRT sessions (two training sessions with the whole group and two with half of the group). The boxes illustrate the 25th and 75th percentiles, bars indicate medians, and circles indicate outliers.

During medical PRT, salivary cortisol levels showed no significant changes in orangutans or bonobos (overall test of the effect of the factor orangutans: P_MCMC_ = 0.435; bonobo: P_MCMC_ = 0.869; Table 4). Furthermore, salivary cortisol levels were independent of sex and age in both species (Table 4). However, in both species cortisol levels differed significantly between sessions (Table 4). Salivary cortisol levels during medical PRT with the whole group were not significant different from levels during training with half of the group (Figure 2).

**Table 4:** Results of the two General Linear Mixed Models of the two subsets for orangutans and bonobos for medical positive reinforcement training with salivary cortisol as response variable (sex, age, and session number are included as fixed effects.

	Estimate	Std. Error	P_MCMC_
**Bonobo**			
Intercept	0.829	0.143	
Sex	0.167	0.197	0.341
Age	0.046	0.088	0.564
Session	0.221	0.044	**0.023**
Pre PRT	0.049	0.094	0.873†
Post PRT	0.018	0.093	
**Orangutan**			
Intercept	1.547	0.214	
Sex	0.214	0.112	0.089
Age	0.089	0.055	0.123
Session	−0.141	0.042	**0.035**
Pre PRT	0.022	0.083	0.395†
Post PRT	−0.076	0.082	

Animal and session ID were included as random intercept terms (MCMC = Markov Chain Monte Carlo)). Bold values indicate P<0.05.

† overall effect of the factor.

## Discussion

Salivary cortisol levels remained unaffected during 20 min. of medical PRT in a group of bonobos and orangutans, respectively. This result was similar for all individuals independent of sex and age. Furthermore, in bonobos the number of individuals participating in a medical PRT session did not affect salivary cortisol levels. When captive orangutans were exposed to potentially stressful events, salivary cortisol increased in comparison to baseline concentrations. This is similar to what has been found in orangutans [58], bonobos [56] and chimpanzees [64] and suggests that salivary cortisol levels provides a biologically meaningful marker of physiological stress in hominoids.

In chimpanzees, Bloomsmith and colleagues showed that during PRT individuals varied in their willingness to participate in training sessions depending on their age and sex [77]. In our analyses we controlled for individual ID. Therefore, we can exclude that certain animals had a disproportionately high impact on the overall result.

Spatial proximity during training may affect stress hormone levels in wild and captive animals [78]. This question was explored by comparing multiple samples obtained at different times within each medical PRT session. If serum cortisol levels increased in the apes as a result of PRT, a corresponding increase in saliva samples would be expected in less than 5 min. [79]–[81]. Therefore, we expected that if there was an effect of medical PRT on salivary cortisol concentration it should be detectable in samples taken after 10 and 20 minutes from the start of the training sessions. However, neither in the samples from orangutans nor in those from bonobos did we find changes in salivary cortisol during the 20 min. medical PRT session. A similar result in salivary cortisol levels during medical PRT was found in baboons [60]. Taken together, these results suggest that the training of these primates to perform behaviours and interactions with humans did not lead to an increase in cortisol levels and, by inference, did not create a stressful situation. However, the absence of a stress response could be also due to the long-term exposure the apes had with the medical PRT. Nevertheless, the results show that in well-trained apes the medical PRT does not induce a stress response in form of an activation of the HPA axis. The effect of human/non-human primate interactions on the well-being of primates was less of a focus in previous studies [22] although the general assumption exists that such interactions have a positive effect on the well-being of the animals [41]. With our study we showed that cortisol excretion in well-trained bonobos and orangutans is not influenced by intensive human/non-human primate interaction during medical PRT. Moreover, by rewarding specific behaviours a closer contact between keepers and animals is established [33], [82]. Taken together, medical PRT in combination with close keeper-animal relationships significantly facilitate the management and handling of primates during complicated management and behaviour problems [42].

Interestingly, the two species differed significantly in salivary cortisol levels across PRT sessions. This result is in contrast to the baboon study by O'Brien et al. [60] which did not find changes in salivary cortisol levels across training sessions. Our findings indicate that the general day-to-day variation in salivary cortisol levels can vary remarkably and therefore it is of paramount importance that baseline samples for comparison for the group and the individual are collected closely in time to the experimental procedure.

In bonobos, we tested the effect of number of animals in a training session. Average salivary cortisol levels were not different between medical PRT with the whole or with only half of the group. Our results suggest that training in a trained group of bonobos does not affect salivary cortisol levels and therefore bonobos were not stressed by this medical PRT. This is important since in general the advantage of having more than one individual for medical PRT is that nonhuman primates are most of the time more relaxed when in a group and furthermore they can learn easier and faster by observing the other individuals [83].

Our results do not indicate that medical PRT in itself improves the welfare of the apes. However, the medical PRT does not increase salivary stress hormones and moreover, it can replace much more invasive procedures, such as anesthesia [1], [14], [34]. By establishing a trustful human-ape relationship (apes showing special parts of the body and tolerance to being touched), medical PRT also allows easier interventions such as wound treatment.

## Conclusions

In orangutans, salivary cortisol increased in stressful situations. However, we did not find significant changes in salivary cortisol during medical PRT in either bonobos or in orangutans, which had years of experience with the training. We conclude that medical PRT is not stressful for bonobos and orangutans which are familiar with PRT, and therefore provides a helpful tool in zoo management of the two species. Furthermore, it seems that in gregarious species like bonobos, the number of animals per session does not influence the stress response. Since daily variations in salivary cortisol occur in both species, it is important that control samples are always taken close to the situation of interest.
